# Clinical Factors and Serum Biomarkers Associated With Major Depressive Disorder in Adolescents With and Without Nonsuicidal Self-Injury: A Comparative Cross-Sectional Study

**DOI:** 10.31083/AP44160

**Published:** 2026-02-02

**Authors:** Shengjiao Zhao, Xiaodan Dong, Jiahui Lu, Yanyan Wu, Ping Fang, Yu Zhang, Yujian Mou, Haiyan Xie

**Affiliations:** ^1^Department of Psychiatry, The Fourth Affiliated Hospital of School of Medicine, International School of Medicine, International Institutes of Medicine, Zhejiang University, 322000 Yiwu, Zhejiang, China

**Keywords:** depression, nonsuicidal self-injury, complement, inflammatory factors, folate

## Abstract

**Background:**

To investigate differences in biological characteristics and factors associated with depressive disorder with or without nonsuicidal self-injury (NSSI) in adolescents.

**Methods:**

This study enrolled adolescents aged 12–18 years, including patients with first-episode depression and healthy controls. According to the Diagnostic and Statistical Manual of Mental Disorders, patients were divided into an NSSI group and a non-NSSI group. Collected data included demographic variables (sex, age, years of education), psychological scale scores (Self-Rating Anxiety Scale [SAS], Self-Rating Depression Scale [SDS]), and biological indicators (folate, immunoglobulins, complement, inflammatory factors). Differences among the three groups were compared using analysis of variance, and correlates of NSSI were explored using regression analysis.

**Results:**

The study included 110 patients with first-episode depression and 55 healthy controls. Among the patients, 57 were classified into the NSSI group and 53 into the non-NSSI group. The following results were obtained: (1) The three groups differed significantly in sex, SAS and SDS scores, and levels of folate, complement 3 (C3), and interleukins 6 and 4 (*p* < 0.05). (2) Female sex and high C3 levels were positively associated with NSSI, whereas age and high folate levels were inversely associated with NSSI. High folate levels were a protective correlate in the non-NSSI group.

**Conclusion:**

Demographic factors such as sex and age influence the development of depressive disorders with comorbid NSSI. In addition, levels of C3 and folate may be related to NSSI behavior in patients with depression.

## Main Points

• Significant Group Differences: Adolescents with first-episode depression and comorbid nonsuicidal self-injury (NSSI) showed distinct demographic, psychological, and biological profiles compared to both depressed patients without NSSI and healthy controls. Key differences included sex distribution, anxiety/depression severity, and levels of folate, complement 3 (C3), and interleukins.

• Identified Correlates of NSSI: Female sex and higher levels of complement C3 were positively associated with the presence of NSSI in depressed adolescents. Conversely, older age and higher folate levels were inversely associated with NSSI.

• Folate as a Potential Protective Factor: Higher folate levels were identified as a protective correlate against NSSI, specifically in the depressed group without self-injury, suggesting a potential role for folate in mitigating self-harming behaviors in adolescent depression.

• Implication of Immune and Inflammatory Pathways: The study highlights the potential involvement of immune system components (specifically C3) and inflammatory factors in the pathophysiology of NSSI comorbid with depression, pointing to possible biological mechanisms underlying this behavior.

## 1. Introduction

After the coronavirus disease 2019 pandemic, the prevalence of depression among children aged 9–15 years has soared from 11.8% to 34.4% [1]. Adolescents in this age group are going through a crucial stage of self-awareness integration and identity formation, and their ability to regulate their emotions is relatively weak. Owing to the lack of emotional stability and inadequate regulation ability, individuals with depression are more prone to atypical symptoms, such as destructive emotions and irritability, and may even resort to extreme behaviors, such as nonsuicidal self-injury (NSSI) or suicide [2]. Individuals with adverse childhood events exhibit greater psychological distress, lower emotional well-being, anhedonia and depression, as well as higher rates of lifetime suicidal ideation and suicide attempts. Studies indicate a close association between adverse childhood events and NSSI in adolescents, with notably increased risks of NSSI across all childhood trauma subtypes [3]. NSSI refers to the intentional and self-initiated act of harming one’s own body tissues without suicidal intent. Currently, the global prevalence of NSSI is on the rise, with an incidence rate ranging from 11.5% to 33.8% [4]. In China, NSSI has been reported in approximately 25% of adolescents [5], seriously hindering their personality development and academic performance. Emotional problems and NSSI are significantly comorbid in adolescents. Research indicates that both depressive disorders and NSSI are key predictors of suicidal behavior in adolescents [6]. Multiple surveys have verified that more than 50% of Chinese adolescents with depression engage in NSSI [7]. When facing adverse childhood events, academic pressure, or peer relationship problems, adolescents with depression often resort to NSSI to regulate their emotions and cognition. Notably, the presence of comorbid NSSI was shown to significantly increase suicide risk among adolescents with depression. Without timely intervention, NSSI may escalate into suicidal behavior, causing serious harm to the physical and mental health of adolescents with depression [8]. Although the aforementioned background highlights the close relationship between depression and NSSI in adolescents and underscores the urgency of further research, the underlying pathological mechanisms of their comorbidity remain poorly understood.

The pathogenesis of depression is closely related to the abnormal upregulation or activation of inflammatory factors, such as cytokines, and the complement system [9, 10, 11]. The immune-inflammatory pathway not only provides a physiological basis for the development of depressive behavior but also reveals the intrinsic connection between inflammatory responses and depressive symptoms [12]. As important cell signaling mediators, cytokines regulate the immune response to internal and external stimuli. This functional class of small proteins includes interleukins (ILs), interferons, chemokines, lymphokines, and tumor necrosis factors [9]. Clinical observations have revealed that IL-6 and white blood cell levels are significantly positively correlated with the severity of depressive symptoms [13, 14]. The underlying mechanism may involve inflammatory responses impairing the function of the frontal lobe, thereby increasing impulsive behavior and suicide risk [15]. Elevated levels of IL-6 are closely associated with somatic symptoms and cognitive dysfunction [16], whereas mice with decreased IL-4 levels exhibited more pronounced depressive-like behaviors in stress tasks [17]. The synergistic effect of anti-inflammatory drugs in the treatment of depression suggests that inflammatory markers may be developed into novel biological targets for assessing the presence of NSSI [18].

As a core component of the innate immune system, the complement system not only mediates proinflammatory signaling but also participates in regulating the development of the central nervous system [19]. Multiple clinical studies have confirmed that the serum levels of complement 3 (C3) and C4 are significantly elevated in patients with depression [15], and C3 expression in the prefrontal cortex is particularly prominent among patients with suicidal tendencies [20]. Conversely, another study showed that the serum level of C3 in patients with first-episode depression was lower than that in patients with recurrent depression and healthy controls, while the serum levels of C4 did not differ significantly among the three patient groups [10]. Given the close connection between the complement system and the inflammatory response, researchers have further explored the role of inflammatory factors in NSSI, but the existing conclusions are also controversial.

In studies on the integration of NSSI, Korean scholars found that the behavioral impulsivity of patients engaging in NSSI was positively correlated with the level of inflammation [21]. Another study demonstrated that the levels of tumor necrosis factor, IL-1, and IL-6 in adolescents engaging in NSSI significantly decreased after treatment, providing strong evidence for the role of inflammatory factors in NSSI [22]. Contrarily, a controlled study in Germany reported no significant differences in the concentrations of IL-6 and other inflammatory factors between patients engaging in NSSI and healthy individuals [23]. Few studies have examined the inflammatory characteristics of Chinese adolescents with depression and NSSI, as well as the underlying pathological mechanism. Therefore, research on the biological basis of depression and its related behaviors (such as NSSI) must take into account the complexity of multi-system interactions, among which metabolic and immune disorders are two important aspects in addition to inflammation.

Neurobiological research indicates that the active form of folate, 5-methyltetrahydrofolate, influences the synthesis of neurotransmitters (dopamine and serotonin) by regulating one-carbon metabolism, with S-adenosylmethionine serving as a key methyl donor. This suggests that folate metabolism disorders may be implicated in the pathophysiology of depression [24]. Multiple controlled studies have confirmed that serum folate levels are significantly lower in patients with depression than in healthy individuals, establishing folate deficiency as an independent risk factor for depression [25]. However, the antidepressant efficacy of folate supplements remains controversial, and their clinical translational value warrants more rigorous causal evidence [26]. Additionally, patients with depression exhibit highly heterogeneous changes in humoral immunity, with multiple studies reporting inconsistent changes in immunoglobulin (Ig) levels (elevated, decreased, or unchanged) [27, 28]. This heterogeneity is a reflection of the complex underlying mechanisms involving multifactorial interactions among psychological, neural, immune, and environmental factors.

In summary, despite evidence substantiating the association of depressive disorders with abnormal folate metabolism, the complement system, Ig levels, and inflammatory factors, the pathological mechanism underlying the comorbidity of depressive disorders with NSSI remains unclear. This study adopts a case-control design to systematically analyze dynamic changes in the levels of immune-neuroregulatory substances in adolescent patients with depression with or without NSSI. The results of this study will shed light on the association between biomarker profiles and NSSI, inform the construction of a risk warning model, and facilitate the early identification of personalized interventions for suicide prevention in adolescents with NSSI comorbid with depression.

## 2. Subjects and Methods

### 2.1 Research Subjects

The research cohort consisted of patients diagnosed with depression who visited the psychiatry outpatient department of the Fourth Affiliated Hospital of Zhejiang University School of Medicine from January 2022 to January 2023. Meanwhile, healthy students undergoing physical examinations were enrolled in the healthy control group. The diagnosis of all patients was confirmed by two psychiatrists at the position of attending physician or above. The participants were informed in detail about the purpose and significance of the research, and they signed the informed consent form. The research cohort was further divided into the depression with NSSI group (here onward referred to as the NSSI group) and the depression without NSSI group (here onward referred to as the non-NSSI group) according to the diagnostic criteria of depression and NSSI in the Diagnostic and Statistical Manual of Mental Disorders, Fifth Edition. The patients who met the inclusion criteria are enrolled in sequence.

Participants were included in this study if they: (1) met the diagnostic criteria for depressive disorders in the Diagnostic and Statistical Manual of Mental Disorders, Fifth Edition; (2) were first-episode patients, aged 12–18 years, without a history of psychotropic drug use; (3) could cooperate in the completion of various questionnaires, psychological assessments, and blood tests; (4) provided informed consent, along with their legal guardians. Participants were excluded if they had: (1) comorbid mental disorders, such as schizophrenia; (2) comorbid physical or organic brain diseases; (3) a history of suicide attempts; (4) a history of alcohol or drug abuse and addiction; and (5) secondary depression caused by organic diseases or drugs.

The control group was randomly selected from students who underwent community health check-ups through a frequency-matching method based on age matching with the research group. The random sampling was conducted using the computer Microsoft Excel 2021 (Microsoft Corporation, Redmond, WA, USA). The sampling interval (k = total number of people in the age group/number of people to be recruited) was calculated, and then starting from a random point, one person was selected every k individuals. Their age and years of education matched those of the research cohort. None of the control group participants had a personal or family history of mental illness. They were all able to cooperate in the completion of various questionnaires, psychological assessments, and blood tests, and they signed the informed consent form.

### 2.2 Research Tools

#### 2.2.1 General Information Questionnaire

A self-designed general information questionnaire was used to collect data such as sex, age, and years of education.

#### 2.2.2 Psychological Assessment Scales

The Self-Rating Depression Scale (SDS), developed in 1964, assesses depressive symptoms through 20 psychological and physiological items reflecting patients’ subjective emotional states [29]. Raw scores are converted to standard scores (raw score ×1.5), and patients’ symptoms are classified according to their raw scores as follows: <50, normal; 50–60, mild; 60–70, moderate; and >70, severe.

The Self-Rating Anxiety Scale (SAS), created by Zung in 1971 [30], evaluates anxiety on the basis of 5 emotional and 15 physical symptoms, using the same scoring method as the SDS.

#### 2.2.3 Blood Sample Collection

Fasting peripheral venous blood samples (5 mL) were collected through venipuncture between 07:00 and 09:00 AM without the use of anticoagulants, and immediately sent to the laboratory for testing. Serum was obtained by centrifugation at 3000 rpm for 15 minutes, divided into aliquots, and stored at –80 °C for subsequent laboratory assays. The serum levels of IL-2, IL-4, IL-6, IL-10, interferon-γ and tumor necrosis factor-α were measured using the ACEA NovoCyte flow cytometer (ACEA Biosciences, San Diego, CA, USA). The assays were performed by the same technician, who was blinded to the samples’ identity and clinical information. Folate testing was performed using the Abbott Alinity i analyzer (Abbott Laboratories, Abbott Park, IL, USA). The levels of Immunoglobulin G (IgG), Immunoglobulin A (IgA), Immunoglobulin M (IgM), C3, and C4 were measured using Beckman Coulter reagents on the IMMAGE 800 protein chemistry analyzer (Beckman Coulter Inc., Fullerton, CA, USA; A: M209282; G: M207375; M: M207377; C3: M207366; C4: M202580).

### 2.3 Data Analysis

Data were analyzed using R software, version 4.3.2 (R Foundation for Statistical Computing, Vienna, Austria). Count data are expressed as n (%). Differences between the three study groups were compared using the chi-square test. Normally distributed quantitative data are expressed as mean ± standard deviation, and intergroup comparisons were made using analysis of variance. Non-normally distributed data are expressed as medians and quartiles, and intergroup comparisons were made using the Kruskal–Wallis test. To identify independent predictors of NSSI and non-NSSI, indicators that were statistically significant (*p* < 0.05) in univariate analyses were subjected to multivariate multinomial logistic regression. SAS/SDS scores were analyzed as continuous variables.

## 3. Results

### 3.1 General Information and Clinical Characteristics

**Fig. 1.**
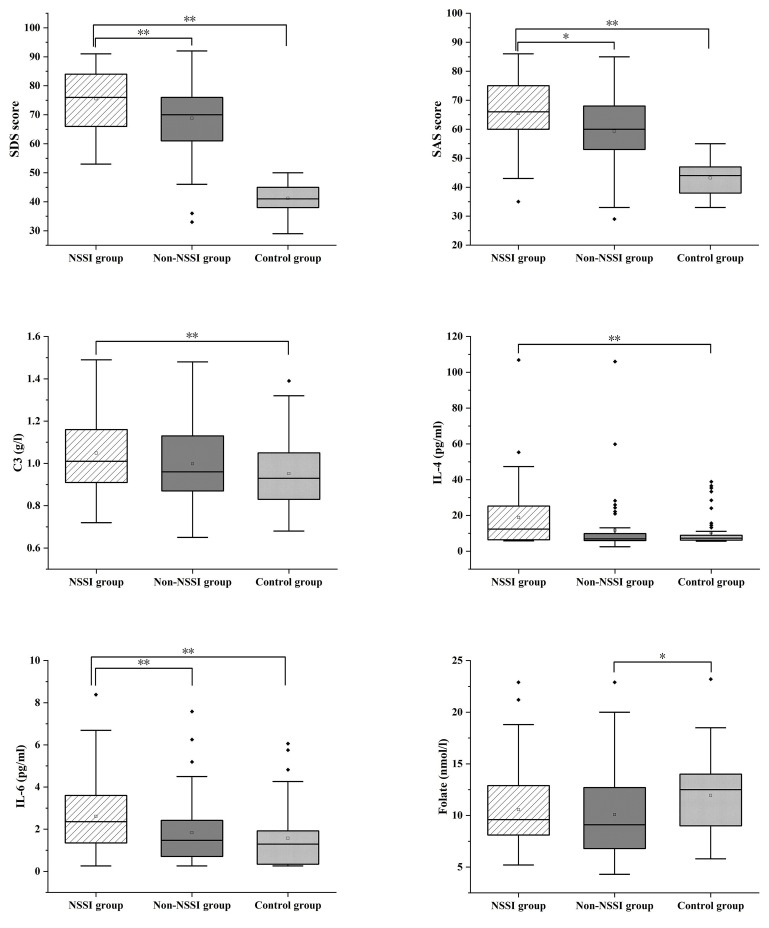
**SDS, SAS, C3, IL-4, IL-6 and Folate were compared in the three groups**. *p < 0.05, **p < 0.01. Notes: The upper whisker extends from the hinge to the largest value no further than 1.5 × IQR from the hinge (where IQR is the Inter-quartile Range, or distance between the first and third quartiles). The lower whisker extends from the hinge to the smallest value at most 1.5 × IQR of the hinge. Data beyond the end of the whiskers are called “outlying” points and are plotted individually.

The sample size estimation formula for case-control studies: $n=\frac{{\left(z_{\alpha }\,\sqrt{2\,\bar{p\,q}}+z_{\beta }\,\sqrt{p_{0}\,q_{o}+p_{1}\,q_{1}}\right)}^{2}}{{\left(p_{1}-p_{o}\right)}^{2}}$. The estimated relative risk value is approximately 4. Among the population of this age group, about 20% have NSSI behavior. The requirement is α = 0.05, β = 0.1, the sample size for each group was approximately 51 people, and the total sample size was 102 people. Considering the possibility of data loss, a dropout rate of 15% was set. Therefore, the required sample size: n = 102 / (1-0.15) = 120. In reality, 10 patients with incomplete data were excluded. The research cohort included 110 patients, of whom 43 were boys and 67 were girls. Among the patients, 57 were classified into the NSSI group and 53 into the non-NSSI group. The control group comprised 55 participants, of whom 30 were boys and 25 girls. General information and clinical characteristics were compared across the control, NSSI, and non-NSSI groups. The three groups significantly differed in terms of sex; SAS and SDS scores; and levels of folate, C3, IL-4, and IL-6 (*p* < 0.05; Table 1). Pairwise comparisons of SDS and SAS scores and levels of C3, IL-4, IL-6, and folate among the three groups are presented in Fig. 1.

**Table 1.** **General information and clinical characteristics**.

Variables		Control group (n = 55)	NSSI group (n = 57)	Non-NSSI group (n = 53)	Statistic	*p*	η ^2^
Sex					7.67	0.02	
	Male	30 (54.55)	17 (29.82)	26 (49.06)			
	Female	25 (45.45)	40 (70.18)	27 (50.94)			
Age (years)		16.00 (14.00, 18.00)	15.00 (14.00, 16.00)	16.00 (14.00, 17.00)	4.36	0.11	0.02
Duration of education (years)		10.00 (8.00, 11.00)	9.00 (8.00, 10.00)	9.00 (8.00, 11.00)	1.05	0.59	0.01
SAS		43.20 ± 5.61	65.39 ± 11.35	59.30 ± 13.14	66.65	0.00	0.46
SDS		41.18 ± 4.89	75.49 ± 10.20	68.79 ± 12.61	176.71	0.00	0.64
Folate (nmol/L)		12.50 (9.00, 14.00)	9.60 (7.84, 12.94)	9.10 (6.65, 12.75)	9.64	0.01	0.05
IgG (g/L)		12.70 (11.00, 14.10)	12.00 (10.70, 13.94)	12.60 (11.25, 13.60)	0.86	0.65	0.01
IgA (g/L)		1.80 (1.43, 2.24)	1.87 (1.34, 2.54)	1.92 (1.44, 2.44)	0.79	0.67	0.01
IgM (g/L)		1.18 (0.89, 1.71)	1.50 (1.06, 1.80)	1.28 (1.02, 1.79)	4.08	0.13	0.01
C3 (g/L)		0.93 (0.83, 1.05)	1.01 (0.91, 1.16)	0.96 (0.87, 1.13)	7.66	0.02	0.04
C4 (g/L)		0.19 (0.16, 0.23)	0.21 (0.17, 0.24)	0.20 (0.17, 0.23)	1.91	0.38	<0.01
IFN-γ (pg/mL)		3.88 (3.88, 14.90)	7.93 (3.88, 16.27)	5.97 (3.88, 13.52)	5.49	0.06	0.02
TNF-α (pg/mL)		26.48 (20.59, 41.31)	32.23 (21.66, 43.14)	25.72 (19.12, 36.19)	3.05	0.22	0.01
IL-2 (pg/mL)		0.12 (0.12, 0.12)	0.12 (0.12, 0.12)	0.12 (0.12, 0.12)	0.72	0.70	0.01
IL-4 (pg/mL)		7.33 (6.20, 8.94)	12.36 (6.42, 25.90)	6.97 (5.93, 9.89)	14.29	0.00	0.08
IL-6 (pg/mL)		1.29 (0.34, 1.92)	2.36 (1.35, 3.62)	1.47 (0.68, 2.49)	18.57	0.00	0.11
IL-10 (pg/mL)		1.85 (1.34, 2.42)	1.78 (1.47, 2.25)	1.75 (1.32, 2.09)	1.00	0.61	0.01

Abbreviation: NSSI, depressive disorder with nonsuicidal self-injury; Control group, healthy control group; SDS, Self-Rating Depression Scale; SAS, Self-Rating Anxiety Scale; C3, complement 3; C4, complement 4; IL-2, interleukin-2; IL-4, interleukin-4; IL-6, interleukin-6; IL-10, interleukin-10; IgG, Immunoglobulin G; IgA, Immunoglobulin A; IgM, Immunoglobulin M; IFN-γ, interferon-γ; TNF-α, tumor necrosis factor-α. Notes: Continuous variables presented as median (interquartile range) are not normally distributed through the Shapiro-Wilk normality test (*p* < 0.05). SAS and SDS scores are presented as mean ± standard deviation, as they followed a normal distribution. The Kruskal-Wallis rank sum test was used to calculate the differences among the three groups of variables. η^2^ values of 0.01 (small), 0.06 (medium), and 0.14 (large) are the standard references.

### 3.2 Factors Influencing Depression With and Without NSSI

Univariate logistic regression analysis was conducted to explore the association between various factors and different clinical manifestations, with the dependent variable being the presence or absence of NSSI in adolescents with depression. Compared with the healthy control group, female sex, SAS and SDS score, and levels of C3, IL-4, and IL-6 were positively associated with the NSSI group, whereas age was inversely associated with it. Similarly, SAS and SDS scores were positively associated with the non-NSSI group, while folate level was inversely associated with it (Table 2). Due to the presence of multicollinearity among independent variables (kappa = 0.310), two variables (SDS and SAS) exhibiting high correlation with others were removed from the multinomial logistic regression model. The results indicated that compared with the healthy control group, female sex and C3 level were positively associated with the NSSI group, whereas age and folate level were negatively associated with it (Table 3). Folate level was negatively associated with the non-NSSI group (Table 4).

**Table 2.** **Results of univariate multinomial logistic regression**.

NSSI group	Coefficients	SE	Z	Wald χ^2^	*p*	OR	95% CI
Sex (Ref = Male)	1.04	0.40	2.61	6.86	0.01	2.82	1.30–6.14
Age (years)	−0.25	0.12	−2.10	4.43	0.04	0.79	0.62–0.98
Duration of education (years)	−0.10	0.12	−0.83	0.68	0.41	0.91	0.73–1.14
SAS	0.20	0.03	6.59	43.37	<0.01	1.23	1.15–1.30
SDS	0.36	0.07	5.32	28.32	<0.01	1.43	1.26–1.64
Folate (nmol/L)	−0.09	0.05	−1.78	3.17	0.08	0.92	0.83–1.01
IgG (g/L)	−0.05	0.08	−0.59	0.34	0.56	0.95	0.82–1.12
IgA (g/L)	0.10	0.27	0.38	0.15	0.70	1.11	0.65–1.88
IgM (g/L)	0.58	0.33	1.73	3.00	0.08	1.78	0.93–3.43
C3 (g/L)	3.24	1.16	2.81	7.87	0.01	25.63	2.66–247.19
C4 (g/L)	5.47	3.79	1.20	2.88	0.10	32.43	1.38–330.51
IFN-γ (pg/mL)	0.01	0.01	0.81	0.65	0.42	1.01	0.99–1.04
TNF-α (pg/mL)	0.01	0.01	0.84	0.70	0.40	1.01	0.99–1.03
IL-2 (pg/mL)	0.59	0.54	1.09	1.20	0.27	1.79	0.63–5.12
IL-4 (pg/mL)	0.05	0.02	2.52	6.33	0.01	1.05	1.01–1.09
IL-6 (pg/mL)	0.47	0.14	3.25	10.54	<0.01	1.60	1.20–2.12
IL-10 (pg/mL)	−0.03	0.10	−0.27	0.07	0.79	0.10	0.81–1.18
Non-NSSI group	Coefficients	SE	Z	Wald χ^2^	*p*	OR	95% CI
Sex (Ref = Male)	0.22	0.39	0.57	0.33	0.57	1.25	0.59–2.65
Age (years)	−0.12	0.12	−1.00	1.00	0.32	0.89	0.70–1.12
Duration of education (years)	0.01	0.19	0.10	0.01	0.92	1.01	0.80–1.28
SAS	0.16	0.03	5.52	30.46	<0.01	1.17	1.11–1.24
SDS	0.38	0.07	4.69	22.00	<0.01	1.36	1.20–1.55
Folate (nmol/L)	−0.12	0.05	−2.38	5.64	0.02	0.88	0.80–0.98
IgG (g/L)	−0.04	0.08	−0.51	0.26	0.61	0.96	0.82–1.13
IgA (g/L)	0.21	0.27	0.77	0.60	0.44	1.23	0.72–2.11
IgM (g/L)	0.37	0.35	1.08	1.16	0.28	1.45	0.74–2.85
C3 (g/L)	1.69	1.18	1.43	2.05	0.15	5.42	0.54–54.62
C4 (g/L)	2.06	3.61	0.57	0.33	0.57	7.86	0.07–632.27
IFN-γ (pg/mL)	−0.00	0.01	−0.23	0.05	0.82	1.00	0.97–1.03
TNF-α (pg/mL)	0.00	0.01	0.05	0.00	0.96	1.00	0.98–1.03
IL-2 (pg/mL)	0.65	0.53	1.23	1.50	0.22	1.92	0.68–5.47
IL-4 (pg/mL)	0.02	0.02	0.75	0.56	0.46	1.02	0.974–1.06
IL-6 (pg/mL)	0.16	0.15	1.07	1.14	0.29	1.18	0.87–1.58
IL-10 (pg/mL)	−0.06	0.11	−0.57	0.32	0.57	0.94	0.76–1.16

Abbreviation: SE, standard error; OR, odds ratio; CI, confidence interval.

**Table 3.** **NSSI group multivariate multinomial logistic regression results**.

Variables	Coefficients	SE	Z	Wald χ^2^	*p*	OR	95% CI
Sex (Ref = Male)	1.18	0.46	2.59	6.71	0.01	3.25	1.33–7.93
Age (years)	–0.24	0.14	–1.990	3.9	0.05	0.79	0.60–1.04
Folate (nmol/L)	–0.16	0.06	–2.63	6.91	0.01	0.86	0.76–0.96
C3 (g/L)	2.48	1.31	1.90	3.78	0.05	11.92	0.92–155.19
IL-4 (pg/mL)	0.04	0.02	1.80	3.25	0.07	1.04	1.00–1.08
IL-6 (pg/mL)	0.25	0.16	1.53	2.33	0.13	1.28	0.93–1.76

**Table 4.** **Non-NSSI group multivariate multinomial logistic regression results**.

Variables	Coefficients	SE	Z	Wald χ^2^	*p*	OR	95% CI
Sex (Ref = Male)	0.47	0.46	1.11	1.23	0.27	1.60	0.70–3.68
Age (years)	–0.17	0.13	–1.27	1.62	0.20	0.85	0.65–1.09
Folate (nmol/L)	–0.16	0.06	–2.71	7.34	0.01	0.86	0.76–0.96
C3 (g/L)	1.33	1.29	1.04	1.07	0.30	3.78	0.31–46.87
IL-4 (pg/mL)	0.01	0.02	0.50	0.25	0.62	1.01	0.97–1.06
IL-6 (pg/mL)	0.06	0.166	0.35	0.12	0.73	1.06	0.77–1.47

## 4. Discussion

This study analyzed the incidence of depressive disorder with comorbid NSSI by sex and age. The results showed that female sex was an independent risk factor for depressive disorder with comorbid NSSI. This finding is consistent with the large-sample study by Sun *et al*. [31], which reported a 3.4-fold higher incidence of NSSI among female adolescents than male adolescents. The possible mechanism underlying this sex disparity may be that women are more likely to adopt externalized coping strategies for negative emotions. Their prominent emotional expression characteristics and higher pain tolerance thresholds may collectively promote NSSI to become a preferred maladaptive strategy for emotion regulation [32]. Multiple logistic regression analysis identified age as a protective factor for NSSI. This is highly consistent with the age-wise trajectory of NSSI development: the behavior often begins at the age of 12–14 years, peaks at 15–17 years, and gradually subsides in early adulthood [33]. The neurodevelopmental perspective provides a plausible explanation for this phenomenon: the asynchronous development of the prefrontal-limbic system during adolescence leads to an imbalance in emotional regulation, manifested by insufficient top-down control from the orbitofrontal cortex and hyperresponsiveness of the amygdala [34]. This neuroplastic window period may form the biological basis for the high incidence of NSSI in adolescents, while the subsequent maturation of these neural circuits may contribute to the observed decline in NSSI behaviors in adulthood.

The study also uncovered significant differences in SAS/SDS scores among the three groups (NSSI group > non-NSSI group > control group), and univariate logistic regression confirmed that the SAS/SDS score was an independent risk factor for depressive disorder with comorbid NSSI. This finding is in line with the longitudinal study by Zhu *et al*. [35], which showed that the persistence of depressive and anxiety symptoms significantly increases the risk of NSSI in adolescents, while symptom relief can effectively reduce the incidence of self-harm behavior. Kim and Yu [36] reported that individuals with high anxiety achieve immediate emotional regulation by engaging in NSSI, which is aligned with the negative emotion accumulation–release model proposed by Baker *et al*. [37]. When the emotional load in the form of depression and anxiety exceeds an individual’s tolerance threshold, NSSI may become the preferred strategy to relieve psychological distress [37].

In terms of biological indicators, levels of IL-6, IL-4, and C3 were identified as significant positive correlates of the NSSI group. However, after adjustment in a multivariate model, only the C3 level remained a statistically significant indicator. Moreover, the levels of C3 and IL-6 significantly differed across the three groups (NSSI group > non-NSSI group > control group). After adjustment in the multivariate model, IL-6 and IL-4 levels did not differ significantly among the three groups. This might be due to the fact that the patients’ disease courses and severity were different when the data were collected, and the time of data collection after the occurrence of emotional events was also different. Secondly, it might be due to the insufficient sample size. The sample size of this study was adequate for univariate analysis, but when multiple variables such as IL-4 and IL-6 were simultaneously included, the model complexity increased, and the statistical power decreased. Beyond analyzing the potential causes for the lack of significant differences in the levels of IL-6 and IL-4 in the model, conducting an in-depth exploration of the theoretical pathophysiological mechanisms of these inflammatory factors will contribute to a more comprehensive understanding of the possible roles they play in NSSI.

Studies have shown that elevated levels of inflammatory cytokines can exacerbate depressive symptoms and increase the risk of NSSI through two possible mechanisms [38]. First, inflammation activation can alter the metabolism of key neurotransmitters in the brain, affecting frontal lobe function and diminishing the ability to inhibit responses, thereby leading to an increase in impulsive behavior and NSSI [21]. Second, the activation of inflammatory cytokines can promote the release of endogenous opioid peptides [39], reducing pain sensitivity. Notably, IL-4 can induce macrophages to release endogenous opioid peptides [40], and this mechanism may underpin the formation and development of NSSI [41]. A study by Li *et al*. [14] discovered that the peripheral IL-6 levels in their 185 study participants increased during the stress period and decreased as the stress period ended, closely mirroring the individual differences in depression susceptibility. Another study compared adolescents who had experienced at least five NSSI events over the past year with a healthy control group, reporting no differences in the levels of IL-6 or C-reactive protein [23]. In future research designs, the exact time of each emotional event (or the time when the researcher induces emotional stimuli) and the subsequent time of blood sample collection should be clearly recorded to more clearly reveal the independent association between emotional events and cytokine levels.

Abnormalities in the complement pathway have been detected in many neurological and neuropsychiatric disorders [42, 43]. In the pathophysiology of these diseases, the complement pathway is inappropriately reactivated, leading to synaptic defects, disruption of neuronal connections, and cognitive impairment [44]. The C3 protein is considered a hub. Research has found that oligodendrocytes can express C3 under stress conditions, thereby participating in the neuroinflammatory cascade reaction and inducing depression [45]. Mechanism studies have shown that in mouse models, synaptic pruning by microglia can be reduced by blocking the HMGB1 (high mobility group box 1)/C1q (complement component 1q)/C3 pathway, thereby improving depressive-like behaviors [46]. Another study found that by knocking down C3 in the hippocampus, the downstream C3aR (complement C3a receptor 1)-GSK3β (glycogen synthase kinase 3 beta) pathway can be inhibited, restoring the activation level of glial cells under inflammatory conditions and alleviating anxiety and depression [47]. In terms of clinical research findings, the levels of C3 and C3a in the plasma of patients with depression are elevated, with higher levels in female patients than in male patients [48]. A study has reported significantly elevated C3 levels in the cerebral cortices of mice exposed to chronic unpredictable stress and mice exhibiting depression-like or suicide-related behaviors [49]. In our study, the serum level of C3 in the NSSI group was elevated, consistent with some previous studies [15, 50]. These results suggest that complement C3 may be a specific biological marker for depression with comorbid NSSI.

This study sheds light on the association between folate levels and depressive disorders. Folate levels exhibited a gradient decline in patients with depressive disorders (non-NSSI group < control group). Further, multivariate regression analysis confirmed folate level as an independent protective factor for depressive disorders. However, the direct association between folate levels and NSSI was weak, with no significant difference between the NSSI and non-NSSI groups. Folic acid may regulate inflammatory responses through DNA methylation and synthesis processes. Insufficient folate supply may be related to the progression of inflammation, or chronic inflammation may lead to increased folate demand. Supplementation of folate may have anti-inflammatory benefits by reducing CRP (C-reactive protein) levels [24, 51]. Previous studies have shown that folate deficiency can lead to depressive symptoms [24, 52, 53] and supplementation of active folate can synergistically enhance the efficacy of antidepressants [54, 55]. This further indicates the complex potential mechanism of multi-factor interaction in the pathophysiology of depression. However, further research on larger and more diverse populations is needed to verify and expand these findings. Moreover, a recent study conducted in South Korea found a significant association between folate levels and fatal and non-fatal suicide attempts during follow-up, suggesting that folate levels can serve as an auxiliary means for predicting suicidal behavior in patients with depression [56]. Overall, folate status assessment is suggested to be incorporated into clinical practice for treating depression and NSSI. This study found no significant differences in Ig levels among the three groups, implying that humoral immune dysfunction is not the core pathophysiological mechanism of NSSI. However, the potential effects of limited sample size on statistical power must be considered before drawing any strong conclusions.

## 5. Conclusion

This comparative study revealed significant differences in clinical profiles and serum biomarkers among depressive adolescents with NSSI, without NSSI, and healthy controls. Female sex and elevated complement C3 levels were identified as significant positive correlates of NSSI comorbidity. Conversely, older age and higher serum folate levels were significant negative correlates of NSSI. A higher folate level was also a protective correlate in depressed adolescents without NSSI. Given the increasing incidence and substantial public health burden of adolescent depression and NSSI, and the current limitations in prevention and treatment, our findings underscore the potential role of immune-inflammatory pathways and metabolic factors in the pathophysiology of NSSI. To advance this field, future research should prioritize multicenter longitudinal studies to elucidate the spatiotemporal dynamics of these biomarkers. The ultimate goal is to develop artificial intelligence-powered, multi-omics prediction models, facilitating a shift in the diagnostic and therapeutic approach for depression with NSSI from a “symptom-driven” paradigm to a more precise “mechanism-targeted” one.

This study has some limitations. First, the cross-sectional design of this study precludes observations of the trends in various factors at different stages of the disease. Second, the sample size was relatively small, preventing the stratification of depression and anxiety by severity and duration. Third, the recruitment of participants from a single center and the local community may introduce selection bias and limit the generalizability of our findings to other regions or healthcare settings. Future studies should recruit a larger and more diverse sample from multiple centers to enhance representativeness and investigate more related factors, especially objective evaluation indicators such as magnetic resonance imaging scans, biochemical tests, and genetic assessments, to further elucidate the mechanisms underlying NSSI behaviors. Such research will eventually result in the formulation of more effective prevention and intervention measures.

## Availability of Data and Materials

The data that support the findings of this study are available from the corresponding author upon reasonable request.
